# CHILDREN’S USE OF INTERACTIVE MEDIA IN EARLY CHILDHOOD - AN EPIDEMIOLOGICAL STUDY

**DOI:** 10.1590/1984-0462/2020/38/2018165

**Published:** 2019-11-25

**Authors:** Sabrina da Conceição Guedes, Rosane Luzia de Souza Morais, Lívia Rodrigues Santos, Hércules Ribeiro Leite, Juliana Nogueira Pontes Nobre, Juliana Nunes Santos

**Affiliations:** a Universidade Federal dos Vales do Jequitinhonha e Mucuri, Diamantina, MG, Brazil.; b Universidade Federal de Minas Gerais, Belo Horizonte, MG, Brazil.

**Keywords:** Child, Mobile applications, Smartphone, Epidemiology, Child development, Criança, Aplicativos móveis, Smartphone, Epidemiologia, Desenvolvimento infantil

## Abstract

**Objective:**

To describe the prevalence of interactive media (tablets and smartphones) use by children aged two to four years old, as well as to characterize this use, and investigate habits, practices, parents’ participation and opinion about their child’s interactive media use.

**Methods:**

A cross-sectional study with 244 parents or legal guardians of children enrolled in daycare centers in a small Brazilian municipality was conducted. A questionnaire based on interactive media use and related habits were applied, and economic level was assessed. Children were divided into three different groups according to media use: Group 1 did not use (n=81); Group 2 uses up to 45 min/day (n=83) and Group 3 uses more than 45 min/day (n=80). Then, they were compared with regard to the sociodemographic variables and media use by the Chi-square test and Student’s t-test.

**Results:**

The prevalence of interactive media use was 67.2%, with a mean time of use of 69.2 minutes/day (confidence interval of 95% - 95%CI 57.1-81.2). The activities most performed were watching videos (55%), listening to music (33%) and playing games (28%). Most parents reported allowing media use in order to stimulate their child’s development (58.4%), accompanying them during use (75.2%), and limiting media time (86.4%).

**Conclusions:**

We observed high interactive media use prevalence. The predominant way of using these devices was marked by parent-child participation. Most parents reported believing in the benefits of interactive media. Passive activities were more frequent, with restricted time of use.

## RESUMO

**Objetivo:**

Descrever a prevalência do uso de mídias interativas (*tablets* e *smartphones*) pelas crianças de dois a quatro anos de idade, assim como caracterizar esse uso, investigar hábitos, práticas, participação e opinião dos pais acerca da sua utilização.

**Métodos:**

Estudo transversal com 244 pais ou responsáveis de crianças matriculadas em creches de um município brasileiro de pequeno porte. Foi aplicado um questionário sobre hábitos de utilização de mídias interativas e investigação do nível econômico. As crianças foram divididas em 3 grupos de acordo com o uso das mídias: grupo 1 - não utiliza; grupo 2 - utiliza até 45 minutos por dia; e grupo 3 - utiliza mais do que 45 minutos por dia. Os grupos foram comparados quanto às variáveis sociodemográficas e de caracterização do uso das mídias por meio dos testes do qui-quadrado e *t* de Student.

**Resultados:**

A prevalência do uso de mídias interativas foi de 67,2%, com tempo médio de utilização de 69,2 minutos por dia (intervalo de confiança de 95% - IC95% 57,1-81,2). As atividades mais realizadas foram: ver vídeos (55%), escutar músicas (33%) e jogar *games* (28%). A maioria dos pais relatou permitir a utilização da mídia para estimular o desenvolvimento do seu filho (58,4%), acompanhá-lo durante o uso (75,2%) e limitar o tempo com a mídia (86,4%).

**Conclusões:**

Observou-se elevada prevalência do uso de mídias interativas. A forma predominante de utilização das mídias envolve conjuntamente crianças e pais, os quais acreditam nos seus efeitos benéficos. Atividades passivas são as mais realizadas, com restrição do tempo de uso.

## INTRODUCTION

The use of interactive media is on the rise among young children. ^1^ ^,^ ^2^ ^,^ ^3^ They have incorporated the use of devices such as smartphones and tablets into their daily routine. This equipment is part of a multimedia system that concomitantly integrates various visual and audio elements (sounds, digital images, compact discs, etc.), which are controlled by computers. The system favors the most effective interaction with a user, as it motivates self-learning and interactive participation.^4^ ^,^ ^5^

Media is being used for a variety of purposes, such as entertainment, leisure, as a distraction in the absence of parents, family communication, and complementary learning through apps.^6^ ^,^ ^7^ ^,^ ^8^ In addition, interactive media devices are practical and can be carried and used anywhere, which facilitates and enhances children and adolescents’ exposure to these devices.^9^

There is a growing number of investigations on this subject, with a significant number of studies focusing on the implications of electronic exhibition media related to the use of television and, more recently, publications based on interactive electronic media,^1^ ^,^ ^10^ ^,^ ^11^ ^,^ ^12^ considering that many questions arise as to the consequences of young children’s continued use of these media and the repercussions on their cognitive, linguistic, social and emotional development.^2^ Experts, associations, and scientific societies around the world^8^ ^,^ ^13^ ^,^ ^14^ seek to establish recommendations on the use of media, but how parents receive and follow these guidelines is still divergent. The literature reports that 75% of two- and three-year-olds exceed recommended use time, ^1^ and parents, when questioned, report partially following the recommendations of the American Pediatric Association regarding media use.^15^ In addition, they are often prompted by doubts and uncertainties about the use of new technology in their families and are concerned about the potential negative effects of technology use on their children’s future.^16^

Thus, there is an explicit need for studies on the way to use media and how much time media is used in order to provide adequate support when offering and limiting the use of this new technology. This study aimed to describe the prevalence of interactive media use by children aged two to four years old, as well as characterize its use, investigate habits, practices, and the participation and opinion of parents regarding its use.

## METHOD

This is a cross-sectional study with probabilistic sampling of the use of interactive media by two- to four-year-old children, regularly enrolled in public and private schools in a small Brazilian city. The study was approved by the Research Ethics Committee of the Universidade Federal dos Vales do Jequitinhonha e Mucuri (UFVJM), under CAAE number 55459916.0.0000.5108.

The study included 244 parents or guardians of children between 24 and 47 months of age, enrolled in the private and public education system of Diamantina, Minas Gerais. The previous sample calculation was performed using the G Power 3.1.9.2 software. For the calculation, the prevalence of mobile interactive media use was estimated at 39%,^17^ with a desired accuracy of 5%, a confidence interval of 95% (95%CI), and a power of study of 80%. Considering the population of 576 children from 24 to 47 months of age, enrolled in the private and public school systems of the municipality studied, an adjustment to the finite number was performed, resulting in 223 children. To this value, 10% was added to compensate for possible losses, reaching a minimum of 245 subjects, who were randomly selected according to the parent or guardians’ availability to answer the questionnaire. All of the parents were eligible to participate in the study. Those who answered the questionnaire incompletely or who did not sign the informed consent form were excluded.

A questionnaire created by the researchers according to information available in the literature about interactive media habits was applied.^2^ ^,^ ^18^ ^,^ ^19^ The instrument addressed questions related to parents’ knowledge of mobile devices, children’s frequency of use, exposure time, age at first use, child’s favorite activities, manner of use, parents’ opinion of their children’s usage.

In order to get information on the economic level of the families, we used the Brazil Economic Classification Criteria 2015 (*Critério de Classificação Econômica Brasil* - CCEB), from the Brazilian Association of Research Companies (*Associação Brasileira de Empresas de Pesquisa* - ABEP). In this instrument, a score is assigned according to property ownership and educational level of the head of the household, and the family’s economic level is graded on an increasing ordinal scale ranging from E to A1.^20^

Initially, the municipal education department and the principals of the participating educational institutions were contacted and the project was presented and discussed. Subsequently, in each educational institution, on pre-established days and times, the questionnaire and the informed consent form were sent home in the backpacks of all 576 children that were of an appropriate age to participate in the study.

As the parents’ adherence was low (n=76; 13%), a shift was made in the educational institutions with regard to the children’s arrival and departure times, in order to inform parents about the benefits and repercussions of the research, as well as the voluntary aspects of participation. With this new approach, 178 more parents (44%) were reached, resulting in a sample of 254. Of this total, ten individuals were excluded because they answered the questionnaire incompletely.

The total interactive media time of the children was evaluated and categorized into three groups, namely: group 1 (n=81) - does not use interactive media; group 2 (n=83) - uses interactive media up to 45 minutes/day; and group 3 (n=80) - uses interactive media more than 45 minutes/day.

The data collected in the questionnaire were analyzed by the Statistical Package for the Social Sciences (SPSS) version 19.0. Categorical variables were described as absolute, and relative frequencies and continuous variables were described by measures of central tendency and dispersion. The normality of distribution of continuous variables was investigated by the Shapiro-Wilk test. To compare groups G1, G2 and G3 regarding sociodemographic and media characterization variables, the chi-square and Student *t* tests were used with a significance level of 0.05.

## RESULTS

**Figure 1:**
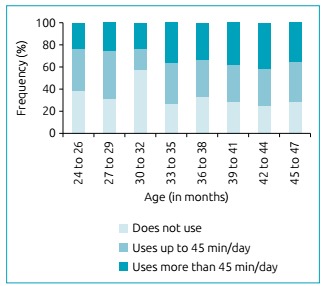
Frequency of interactive media use by age group.

**Figure 2:**
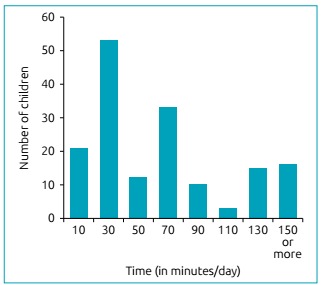
Interactive media usage.

A total of 244 children with ages ranging from 24 to 47 months participated in the research. The average age was 35.5 ± 5.6 months. Of these, 124 (50.8%) were female and 164 (67.2%) had access to interactive media at home. The average use time of the media was 69.2 minutes, ranging from five to 480 minutes/day, with a median of 45 minutes. The frequency and use time of the media, according to age group, can be seen in Figures 1 and 2, respectively.

The prevalence of media use among children aged 24 to 35 months was 63.2%; and in children from 36 to 47 months, it was 70%. Smartphones were children’s most commonly used media: 57.3% from 24 to 35 months, and 59.2% from 36 to 47 months, followed by tablets, used by 21.4 and 25% of children, respectively. Children aged 24 to 35 months had a mean use time of 70.8 minutes/day (95%CI 51.2-90.1) and children aged 36 to 47 months had a mean time of 69.5 minutes/day (95%CI 53.1-85.8), which did not differ statistically (p=0.91).

The reasons why parents allowed children to use the media were: to distract their children in public (n=25; 15.3%), to distract them at home (n=83; 50.9%); and to stimulate their development (n=97; 59.5%).

**Figure 3:**
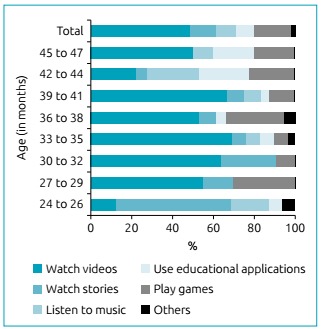
Children’s preferred activity when using interactive media, categorized by age group in months.

Chrildren’s preferred activity according to age can be seen in Figure 3. The characteristics of the three groups in relation to sociodemographic variables and ways of using media are described in Table 1. The average age of the groups did not differ statistically, as follows: 34.8 ± 5.5 (G1), 35.3 ± 5.9 (G2) and 36.3 ± 5.4 (G3).

**Table 1:** The characterization of groups according to sociodemographic variables and ways of using interactive media.

Variable		Group 1 Does not use media n (%)	Group 2 Uses up to 45 min/day n (%)	Group 3 Uses more than 45 min/day n (%)	p-value
Gender	Male	38 (46.9)	40 (48.2)	42 (52.5)	0.75
Age	24 to 35 months	43 (53.1)	41 (51.2)	34 (46.6)	0.39
	36 to 47 months	38 (46.9)	42 (52.5)	46 (57.5)	
Economic level	A and B	4 (5.0)	25 (30.1)	30 (38.5)	<0.001
	C, D and E	76 (95.0)	58 (69.9)	48 (61.5)	
Maternal education level	Only elementary school	33 (46.6)	16 (20)	11 (14.1)	<0.001
	High school	34 (46.6)	37 (46.3)	43 (55.1)	
	Higher education	6 (8.2)	27 (33.8)	24 (30.8)	
Media used	Smartphone	--------------	72 (86.7)	67 (83.8)	0.44
	Tablet	--------------	16 (19.3)	42 (52.5)	<0.001
Watch videos	Yes	--------------	53 (63.9)	66 (82.5)	0.007
Listen to music	Yes	--------------	29 (34.9)	39 (48.8)	0.035
Watch stories	Yes	--------------	24 (28.9)	38 (47.5)	0.015
Play games	Yes	--------------	26 (31.3)	38 (47.5)	0.074
Use educational apps	Yes	--------------	15 (18.1)	31 (38.8)	0.003
Look at photos and make videocalls	Yes	--------------	6 (7.3)	3 (3.8)	0.225
Uses media with parents	Yes	--------------	66 (79.5)	56 (70)	0.16
Parents limit use time	Yes	--------------	63 (90)	68 (85)	0.35
Parents stimulate the children during use	Yes	--------------	53 (80.3)	67 (88.2)	0.19
Parents limit inappropriate content	Yes	--------------	56 (93.3)	66 (88)	0.29
Parents believe in the benefit of the media	Yes	--------------	46 (65.7)	53 (69.7)	0.60

## DISCUSSION

The prevalence of interactive media use in children aged two to four years old in the municipality was 67.2%, with smartphones being the most commonly used media, followed by tablets. An epidemiological study with parents and children in the US revealed that among children 0 to 2 years old, 42% used tablets and 37% used smartphones, which was lower than the percentages found in children aged three to five, 56 and 46%, respectively.^21^ In infants two years of age and younger in Singapore, the prevalence of mobile device use found was 30.5%. ^11^ Common Sense Media, in 2013,^22^ pointed to a growth in media use by children in the United States aged 8 and under, with a prevalence of 52% in 2011, and 75 % in 2013. It is possible to observe a high rate of early childhood interactive media use, and its increase over the years. Although the Brazilian children investigated reside in a place with different sociodemographic characteristics from the aforementioned places, they have a similar media use, showing that this is a worldwide phenomenon, like television, which is little affected by geographical barriers. ^11^ ^,^ ^12^ ^,^ ^21^

In the present study, a statistically significant difference was observed between the groups regarding socioeconomic variables. It was measured by the ABEP classification and maternal education, with an association between lower income and educational level and not using media (Table 1). Thus, 51.2% of children from the lowest income levels (C2, D and E) used media, which is a lower proportion than that of children from income levels A, B and C1 (85.7%). The literature indicates that lower socioeconomic levels are related to higher media use.^1^ ^,^ ^7^ Common Sense Media, in 2013,^22^ showed that media use increased in children with low socioeconomic status, which increased by 22% in 2011 to 65% in 2013, a value similar to that found in the Brazilian children of this study. Regardless of the family’s economic condition, there has been a growing use of tablets and smartphones in children from the United States. However, there is still a difference between socioeconomic levels, and while 65% of the most affluent children have smartphones at home, only 20% of disadvantaged children have this kind of device.^22^

Media usage did not vary by age (Table 1). The literature is controversial about this topic. There are studies where age factor is related to media use.^7^ Also, there are contradictory studies that show the lack of influence of age on the frequency of media use in childhood.^3^ ^,^ ^15^ However, the way media is used is influenced by age, and more elaborate activities are observed in older children.^15^ Interactive media usage time is very variable, with values from 15 minutes/day to two ­­hours/­day.^1^ ^,^ ^3^ ^,^ ^15^ ^,^ ^21^ ^,^ ^22^ ^,^ ^23^ ^,^ ^24^ ^,^ ^25^ The Brazilian Society of Pediatrics (*Sociedade Brasileira de Pediatria* - SBP) in 2016,^14^ recommended that media use time be proportional to the age of the child and their neuropsychic skills. Moreover, children from two to five years old should not spend more than an ­hour/­day in front of a screen. The American Academy of Pediatrics (AAP), also in 2016, ^8^ discouraged children under 18 months to use media, except for video calls. Parents of infants 18 to 24 months old, who wish to introduce the technology, are recommended to use it in conjunction with high quality activities. For children older than two years old, limits are recommended regarding the use of interactive media for one hour/day or less. It was observed that 44% of the children investigated in this study exceeded the time recommended by the SBP and AAP (Figures 1 and 2) and 19% of children spend more than two hours/day in front of screens, which is worrying, considering that the benefits and harms of media are not yet well understood in the literature. ^8^ ^,^ ^14^ ^,^ ^15^ ^,^ ^23^

The activities performed by children using media were: watching videos (55%), listening to music (33%), playing games (28%), watching stories (28%), using educational applications (21%) and others, like looking at photos and making video calls (4%). According to age (Figure 3), “watching videos” was the most frequent favorite activity in all age groups except for children 24-26 months who prefer listening to music. After 26 months of age, activities such as playing games and watching stories were observed. These findings corroborate research findings in French children aged five to 40 months, in which infant preferences were viewing photos (78%) and videos (68%), listening to music (5%) and games (4%), activities influenced by the age of the infant.^15^ Activities where the child only views content (videos, photos, stories) on handheld devices do not favor the child’s interaction with such devices, because it makes the use of interactive media similar to television.^3^ ^,^ ^15^

When asked how their child uses interactive media, the majority of parents (75.2%) reported accompanying their children during use. This is similar to data found in families with American children of the same age - 76% of children use media accompanied by their mothers -^23^ and in Chinese families - 64.4% of parents spent at least 1 hour a day with their children while using the media.^7^ The presence of parents is a factor emphasized in the literature in that they enhance the benefits of media, develop motor, cognitive and language skills, and improve parent-child interaction.^2^ ^,^ ^7^ What is important is how one uses media and not the technology in and of itself.^2^

In the present study, 86.4% of parents reported limiting their children’s use of interactive media. This is essential, considering that the preschool phase is a critical period for child development. It is a phase in which children establish lifelong health habits. In this phase, there is greater parental control over the health behavior of their children.^26^ Because the beneficial effects of media use are not well established in the literature, there is a need to infer parents’ opinion on the subject in order to set standards. In the study by Hamilton et al.,^26^ a few factors were identified that led parents to limit media usage time to less than one hour/day. Among them, parents’ need to establish their role as a parent is emphasized. In addition, the study shows that the opinions of spouses, partners and friends also influence the limitation of media use. The literature reveals that restriction of screen time to less than two hours/day is mainly performed by women and parents who spend less time in front of a screen.^25^ It is clear, therefore, that the investigated parents adopt desirable practices regarding media use. Possible justification lies in the fact that the majority (67.7%) consider the developmental effects of their children to be beneficial.

Smartphones and tablets were used most by the children of this study, and a statistically significant difference was observed between groups G2 and G3 in relation to tablets (Table 1). Studies such as that of Prince et al.^27^ show that the use of tablets can enhance fine motor skills in children aged two to three years old. The French Academy of Sciences, in 2013,^13^ pointed out that the visual and tactile functions of tablets can be useful for the sensory and motor development of children, increasing their learning. However, they may pose risks in that they keep children from doing physical, social and emotional activities related to their age. Lin et al.,^28^ report that activities using tablets can lead to improper physiological changes in the wrist and finger joints. The literature is still in controversy regarding the effects of these media on child development and, therefore, longitudinal studies are needed to monitor the use of media and their effects in a controlled manner.

The present study reveals groundbreaking data on the use of interactive media in early childhood, which is relevant in contemporary times, as the use of interactive media is behind only TV, surpassing media such as computer and video games^1^ ^,^ ^11^. Easy access to interactive media, coupled with the ease of transport and use of these devices, has favored the spread of this type of technology in all age groups, especially in early childhood.

One of the limitations of this study is the possible memory bias, since data collection was performed with a questionnaire answered by parents. However, this way to measure media time is the most widely used in the literature, whether it be in person^11^ ^,^ ^26^ or virtually.^15^ ^,^ ^23^ ^,^ ^26^ Another limitation to be considered is that sample selection was based on parents’ willingness to answer the questionnaire, which may disrupt the probabilistic sampling process, as those parents who responded could be more connected to the topic at hand. Nevertheless, the sample included 42.4% of the target population, which shows an average adherence to the project.

This study confirms the hypothesis that young children are already attracted to interactive media. Strategies are needed in order to reflect on the rapid growth of regular interactive device practices in the early years of childhood until their future impacts on child health and development are known
